# Deciphering Antibody Responses to Orthonairoviruses in Ruminants

**DOI:** 10.3390/microorganisms9071493

**Published:** 2021-07-13

**Authors:** Julia Hartlaub, Markus Keller, Martin H. Groschup

**Affiliations:** Institute of Novel and Emerging Infectious Diseases, Friedrich-Loeffler-Institut, Suedufer 10, 17489 Greifswald, Insel Riems, Germany; julia.hartlaub@fli.de (J.H.); markus.keller@fli.de (M.K.)

**Keywords:** HAZV, DUGV, NSDV, CCHFV, cross-reactivity, serology, specificity

## Abstract

Antibody cross-reactivities between related viruses are common diagnostic challenges, resulting in reduced diagnostic specificities and sensitivities. In this study, antibody cross-reactions between neglected members of the genus *Orthonairovirus*—Hazara (HAZV), Dugbe (DUGV), and Nairobi sheep disease orthonairovirus (NSDV)—were investigated. Mono-specific ovine and bovine sera following experimental infections as well immunization trials with HAZV, DUGV, and NSDV were tested in homologous and heterologous virus-specific assays, namely indirect ELISAs based on recombinant N protein, indirect immunofluorescence assays (iIFA), and two neutralization test formats (plaque reduction neutralization test (PRNT) and micro-virus neutralization test (mVNT)). The highest specificities were achieved with the ELISAs, followed by the mVNT, iIFA, and PRNT. Cross-reactivities were mainly observed within the Nairobi sheep disease serogroup–but surprisingly, HAZV antibodies in PRNT did also neutralize NSDV and DUGV. In conclusion, we recommend ELISAs and mVNTs for a discriminative diagnostic approach to differentiate between these antibodies. NSDV antisera were also used in serological assays for the detection of antibodies against the human pathogen Crimean-Congo hemorrhagic fever orthonairovirus (CCHFV). Interestingly, all CCHFV ELISAs (In-house and commercial) achieved high diagnostic specificities, whereas significant cross-reactivities were observed in a CCHFV iIFA. Previously, similar results were obtained when analyzing the HAZV and DUGV antisera.

## 1. Introduction

The genus *Orthonairovirus* (order *Bunyavirales*) includes predominantly tick-borne arboviruses, which are further classified into seven serogroups [1,2]. Two of these serogroups encounter viruses with special impact for human and animal health, respectively. The Nairobi sheep disease serogroup assigns Nairobi sheep disease orthonairovirus (NSDV), Dugbe orthonairovirus (DUGV), and Kupe orthonairovirus (KUPV). The Crimean-Congo hemorrhagic fever serogroup includes Crimean-Congo hemorrhagic fever orthonairovirus (CCHFV) and Hazara orthonairovirus (HAZV) [2]. NSDV can cause a fatal hemorrhagic gastroenteritis in small ruminants and is therefore an OIE notifiable disease [3]. In parallel, CCHFV can induce a severe hemorrhagic fever in humans and is part of the WHO R&D list for priority diseases [4]. Furthermore, also NSDV and DUGV can lead to mild febrile illnesses in humans [5,6,7]. In contrast, HAZV is thought to be non-pathogenic for humans [8].

Ruminants are susceptible hosts for members of both serogroups, either as the primarily affected animals, actually suffering from the disease (NSDV: small ruminants), or as subclinical hosts in the tick-vertebrate-tick cycle (NSDV: cattle; DUGV and CCHFV: ruminants) [9,10,11]. Whereas the role and importance of the first group is obvious—considering animal health, economic loss, and trading barriers—the second group should not at all be neglected. Ruminants can still be a source of infection for humans, when these get in contact with viremic blood of infected livestock. In addition, ruminants are suitable sentinels for virus circulation, as the presence of specific antibodies is an indicator for the actual virus occurrence [12]. Therefore, seroprevalence data encountering the current distribution and spread of these viruses can contribute to a better risk assessment for viral infections in both humans and ruminants.

For such serological investigations (epidemiological surveys and outbreak characterizations) it is crucial to assess the specificity of currently used diagnostic assays—especially due to the overlapping distribution range of these viruses primarily in Africa and Asia. Although these viruses are members of distinct serogroups, a distant serological relationship between the CCHFV and NSDV serogroup is presumed [13,14,15,16]. Furthermore, genetic analyses have revealed that these viruses actually built up one single genogroup and therefore, close antigenic similarities are not surprising [17]. In consequence, cross-reactive antibodies might lead to false-positive results. To verify the correct serological discrimination of these antibodies by diverse serological assays, mono-specific antisera against these related viruses are essentially needed. Recently, experimental infection as well as immunization studies in sheep and bovines with HAZV, DUGV, and NSDV have been carried out in order to obtain specific antisera originated from the species of interest [18,19,20]. Few studies concerning cross-reactivities within the genus *Orthonairovirus* have been published until the 1980s and are referred to in the majority of recently published articles [13,14,15]. However, these reports are fragmentary and in parts contradictory. Therefore, the transfer of former observations to data interpretation within new studies may be misleading. So far, mainly complement fixation, immunoprecipitation, immunofluorescence, and hemagglutination inhibition tests have been carried out, but nowadays primarily ELISAs are performed routinely. Additionally, only hyperimmune mouse or rabbit sera (and hyperimmune ascitic fluids) were used to evaluate the antigenic relationships between these viruses. No detailed studies were carried out using post-infection sera from ruminants, which are common target species in monitoring programs. Thus, the antibody profiles of our ruminant sera might therefore better mirror the antibody status of field samples than hyperimmune mouse sera.

For this presented report, we investigated, whether antibodies against HAZV, DUGV, and NSDV cross-react to each other in the new assays, which were developed during the animal experiments. Namely N protein-based ELISAs, indirect immunofluorescence assays, and two neutralization test formats (mVNT, PRNT) were established for the detection of HAZV, DUGV, and NSDV antibodies, respectively. Their sensitivity was already compared during the actual infection studies, as some tests did not/or later detect seroconversion of individual animals. The subsequent specificity studies involving multiple antisera against related viruses will further contribute to a more profound validation of results obtained with these assays.

Furthermore, we have already evaluated the specificity of three CCHFV ELISAs with the HAZV and DUGV sera and have demonstrated an overall high specificity of these assays. In contrast, limited cross-reactivities were observed in an indirect CCHFV immunofluorescence assay [18,19]. In order to extend these observations, we have now also included antisera against NSDV into these analyses. These results revealed whether antibodies directed against NSDV lead to positive results in the CCHFV diagnostic assays.

## 2. Materials and Methods

### 2.1. Viruses and Cells

Hazara orthonairovirus (prototype strain JC280) was kindly provided by Ali Mirazimi, National Veterinary Institute, Sweden. Dugbe orthonairovirus (prototype strain IBAR1792) and Nairobi sheep disease orthonairovirus (Ganjam prototype strain IG619) were a kind gift from the World Reference Center for Emerging Viruses and Arboviruses, University of Texas Medical Branch, Galveston, USA. All viruses were grown on SW13 cells, which were also provided by Ali Mirazimi. Methods for quantification of viral titers were conducted following similar protocols for all three viruses (TCID_50_ endpoint-titration and plaque assay). For immunofluorescence assays, Vero E6 cells (Collection of Cell Lines in Veterinary Medicine, Friedrich-Loeffler-Institut, Germany) were utilized.

### 2.2. Sera

Several experimental infection studies involving sheep and cattle have been carried out by our group during the last years with HAZV, DUGV, and NSDV. Furthermore, one sheep and one calf were repeatedly immunized with formalin-inactivated HAZV, DUGV, and NSDV virus stocks, respectively. Hence, also two specific HAZV ruminant antisera are available, although the infected animals did not show seroconversion following the HAZV challenge. In Table 1 all animal sera, which were involved in this study are listed. Detailed information concerning infection doses and immunization schemes can be found in the original articles [18,19,20].

### 2.3. Diagnostic Assays

As described before, recombinant N proteins were expressed in *E. coli* and used as specific antigens for the development of indirect ELISAs. Indirect immunofluorescence assays were carried out utilizing infected Vero E6 cell cultures. Two neutralization test formats were established, both employing SW13 cell monolayers (micro-virus neutralization test (mVNT) and plaque reduction neutralization test (PRNT)). All protocols were carried out as described before [18,19,20]. Table 2 shows which serum dilutions were used for the individual assays and under which conditions sera were scored positive. All sera, including sera prior to infection or immunization were analyzed once with all assays. Positive scores are based on two independently obtained positive results in the same assay format respectively. Mean neutralizing titers were calculated for the mVNT and PRNT respectively. 

Furthermore, all NSDV antisera were run in three different CCHFV ELISA systems and one indirect immunofluorescence test. The multispecies double antigen ELISA (IDVet, Grabels, France) was applied according to the manufacturers’ instructions. An in-house ELISA based on recombinant N protein and a species adapted protocol for the commercial human Vector-Best IgG ELISA (Vector-Best, Novosibirsk, Russia) as well as a species adapted protocol of an immunofluorescence assay (Euroimmun, Luebeck, Germany) were used as described before [21].

## 3. Results

### 3.1. Serological Cross-Reactions between HAZV, DUGV, and NSDV

#### 3.1.1. Indirect ELISA Based on Recombinant N Protein

Within our previous work, species-specific indirect ELISAs were developed for HAZV, DUGV, and NSDV and negative cut-offs were determined using 100 bovine or ovine German reference sera respectively (Cut-off = mean + 3 × standard deviation). Corrected OD values were calculated based on the percentage of the sample compared to the corresponding positive control (immunized sheep and calf). The individual cut-off values varied from 16.2% to 37.5% for these ELISAs, as the protocols differed in some aspects e.g., the dilution factors of the secondary antibody and moreover, the positive controls, which serve as reference sera for the OD value calculations, were variably pre-diluted.

None of the infected animals was tested positive with a heterologous antigen, implying an overall high specificity of these assays. However, the DUGV-immunized calf was tested positive not only with the DUGV antigen, but also with the NSDV antigen (DUGV-IMMU-calf-1: 38.2% > NSDV ELISA cut-off: 26.0%).

#### 3.1.2. Indirect Immunofluorescence Assays (iIFA)

The sera of immunized animals did show high non-specific background reactions, most likely due to antibodies raised against cell debris or FCS (fetal calf serum) during the immunization. Therefore, the cross-reactivities could not be reliably evaluated and only post infection sera are presented here. All these post-infection sera led to distinct positive or negative results.

**Figure 1:**
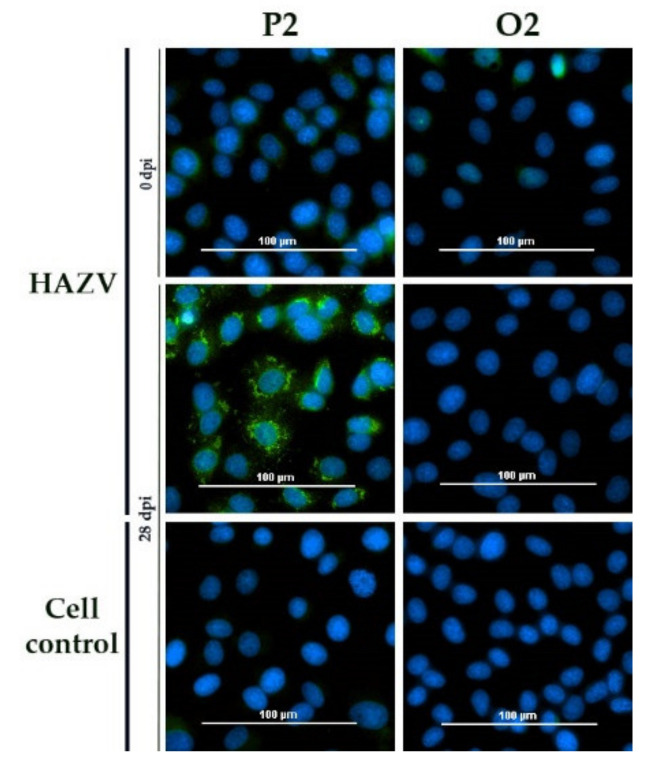
HAZV iIFA: The post infection serum of sheep P2 led to a specific staining of HAZV-infected Vero E6, whereas the (NSDV) mock control O2 did not test positive. Sera prior to infection as well as post infection sera tested on non-infected Vero E6 cells, did not reveal a specific staining.

For HAZV, all sera were tested negative apart from sheep P2, which actually yielded the highest titer of NSDV antibodies (Figure 1).

**Figure 2:**
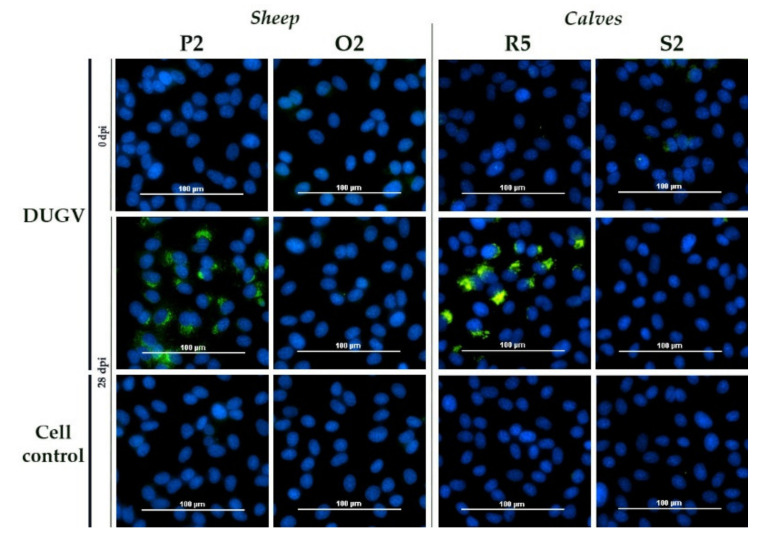
DUGV iIFA: The serum of sheep P2 (NSDV) and calf R5 (NSDV) led to a specific staining of DUGV-infected Vero E6, whereas the mock controls (sheep O2, calf S2) did not test positive. No non-specific signal was detected for pre-challenge serum.

**Figure 3:**
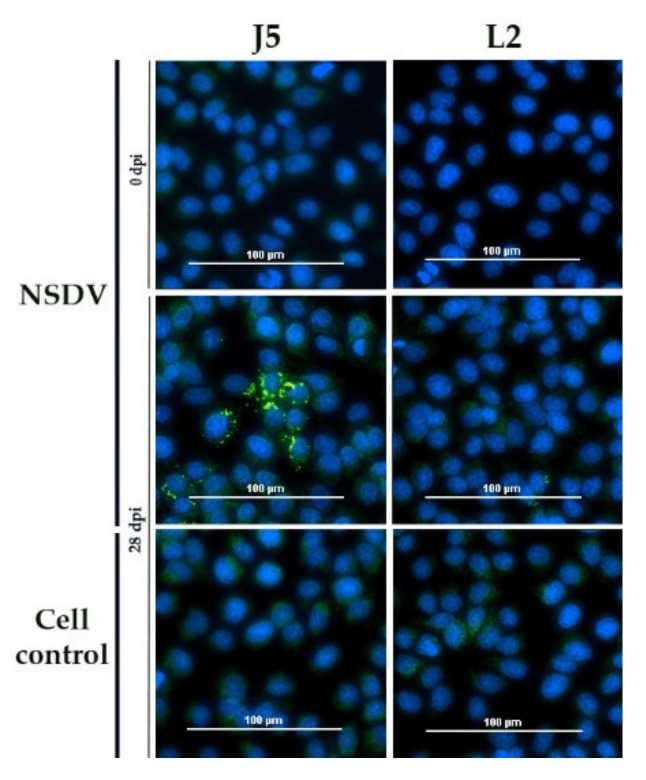
NSDV iIFA: The serum of calf J5 led to a very weak, but distinct staining of NSDV-infected Vero E6, whereas the mock control L2 did not test positive. All other control wells (serum prior to infection, cell controls post infection) were negative.

In contrast, infected NSDV sheep and calves were tested positive with the DUGV iIFA (example sheep P2 and calf R5, Figure 2) and, vice versa, two infected DUGV calves were also weakly positive on NSDV-infected cells (example calf J5, Figure 3).

#### 3.1.3. Micro-Virus Neutralization Assay (mVNT)

**Figure 4:**
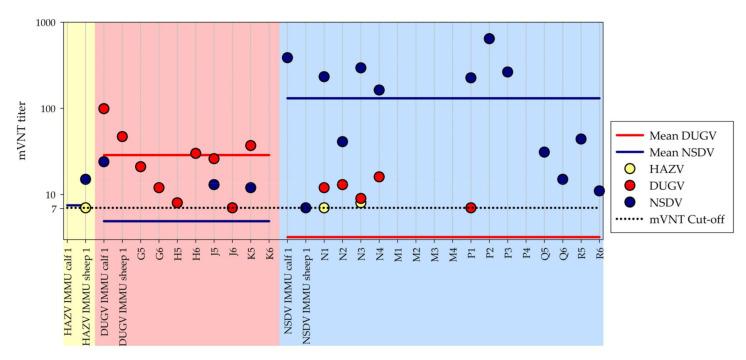
Cross-testing mVNT results: This figure visualizes the mVNT titers for the respective end point sera for all infected and immunized animals. The yellow, red, and blue sections show the results for the ruminants inoculated with HAZV, DUGV, and NSDV, respectively. The great majority of infected and immunized animals revealed higher mVNT titers against the homologous virus than the heterologous viruses. Mean mVNT titers were clearly higher for DUGV in the red section and for NSDV in the blue section, represented by the red and blue line, respectively. For HAZV only very weak mVNT titers were seen for some NSDV sheep.

All sera were tested in biological and technical replicates in the micro-virus neutralization test (Figure 4).

In the HAZV mVNT, only very low neutralizing titers (between 1/5 and 1/10) were seen for the immunized HAZV sheep and the surviving NSDV sheep (2 out of 6 ≥ 1/7). 

In the DUGV mVNT, not only DUGV-immunized and infected animals were tested positive, but also the NSDV-infected sheep.

In the NSDV mVNT, in addition to the NSDV-infected sheep and cattle, also the immunized HAZV sheep, the immunized DUGV calf and two infected DUGV calves were tested positive.

When comparing the mVNT titers, the titers for the homologous viruses are significantly higher than for the heterologous viruses (e.g., sheep N1 NSDV: 1/233 vs. DUGV: 1/12). However, this difference was slightly lower for weak positive sera (e.g., calf J5: DUGV: 1/26 vs. NSDV 1/13).

All sera prior to infection or immunization as well as sera of the control animals were clearly negative. Detailed results for each animal are listed in the Supplementary Material, Table S1.

#### 3.1.4. Plaque Reduction Neutralization Assay (PRNT)

**Figure 5:**
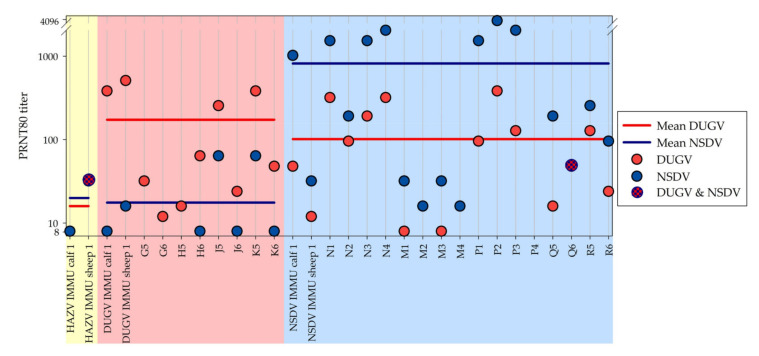
DUGV and NSDV PRNT results: This figure visualizes the PRNT_80_ titers for the respective end point sera for all infected and immunized animals. The yellow, red, and blue sections show the results for the ruminants inoculated with HAZV, DUGV, and NSDV, respectively. The great majority of infected and immunized animals revealed higher PRNT_80_ titers against the homologous virus than the heterologous virus from the same serogroup. Mean PRNT_80_ titers were clearly higher for DUGV in the red section and for NSDV in the blue section, represented by the red and blue line, respectively.

All sera were tested with the HAZV, DUGV, and NSDV PRNT (Figure 5). 

For HAZV, only one serum revealed weak neutralizing antibodies (HAZV-IMMU-calf-1, PRNT_80_ = 1/8) and even the HAZV-immunized sheep did not test positive although repeatedly tested.

In the DUGV PRNT, not only the DUGV-immunized and infected animals were tested positive, but also the HAZV-immunized sheep, NSDV-immunized animals, and the majority of the NSDV-infected animals.

In the NSDV PRNT, the HAZV- and DUGV-immunized animals were tested positive as well as the DUGV-infected cattle.

All sera prior to infection or immunization as well as sera of the control animals were tested negative with all of these assays. In general, antibody levels were significantly higher for the homologous than for the heterologous virus (e.g., sheep P3 PRNT_80_ NSDV: 2048 vs. PRNT_80_ DUGV: 128). However, this difference was not that clear for weak positive sera (e.g., calf R5 PRNT_80_ NSDV: 256 vs. PRNT_80_ DUGV: 128). Several sera would have led to false positive results when individually tested against heterologous viruses only. Two animals (HAZV-IMMU-sheep-1, calf Q6) revealed the same PRNT_80_ titers for NSDV and DUGV respectively. The serum of the HAZV-immunized sheep clearly neutralized DUGV as well as NSDV, even though these viruses are members of another serogroup. Detailed results can be found in the Supplementary Material (Table S2).

### 3.2. Cross-Reactions with NSDV Antisera in CCHFV Assays 

All post NSDV immunization or infection sera were tested with currently used CCHFV serological assays to investigate putative cross-reactivities (Table 3). All sera were negative in the Vector-Best ELISA, IDVet double antigen ELISA, and in the FLI CCHFV in-house ELISA with one sole exception as surprisingly, the immunized calf (NSDV-IMMU-calf-1) was tested positive with all of these assays.

**Figure 6:**
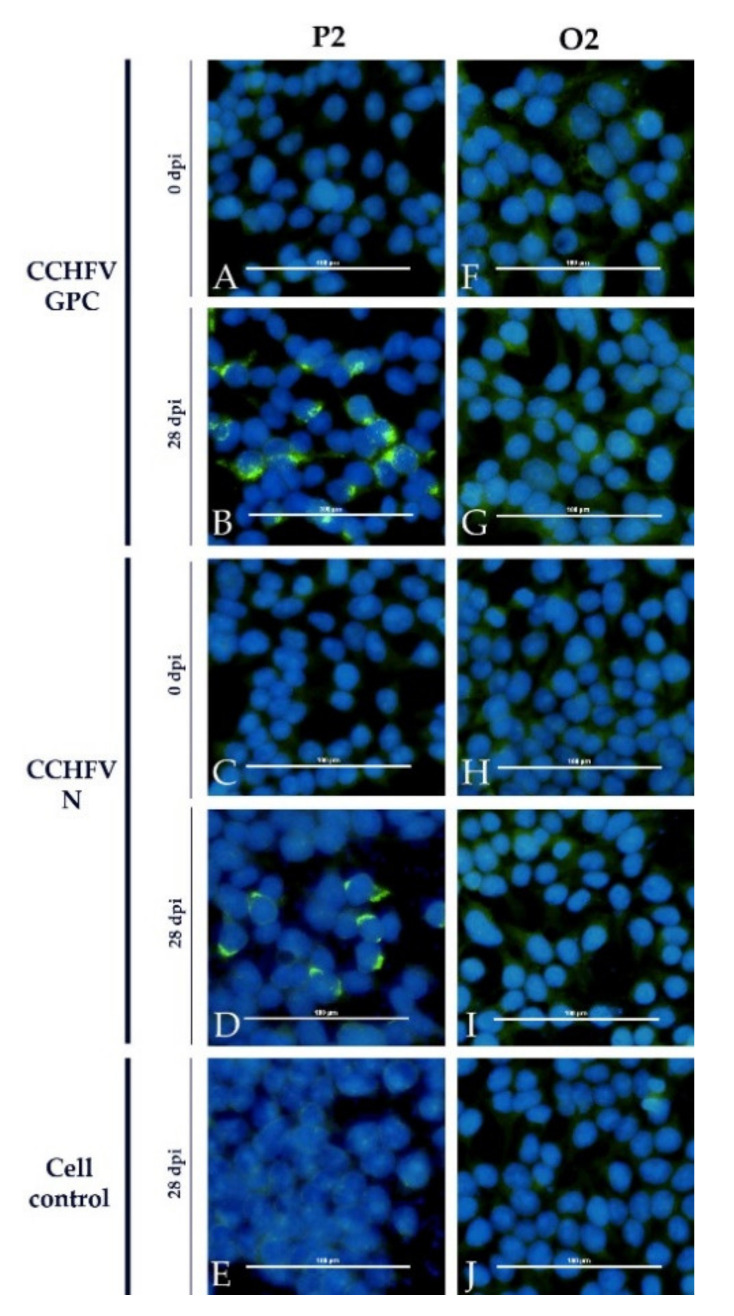
Euroimmun iIFA with sheep NSDV antisera: The results obtained with the serum of an infected sheep (P2) are presented as an example together with the results for the control sheep (O2). A specific staining is demonstrated for cells expressing CCHFV GPC (**B**) as well as CCHFV N (**D**) for P2. All controls (cell control: (**E**); serum prior to infection: (**A**,**C**)) did not lead to a positive signal. The control sheep O2 did not test positive in any condition (**F**–**J**).

**Figure 7:**
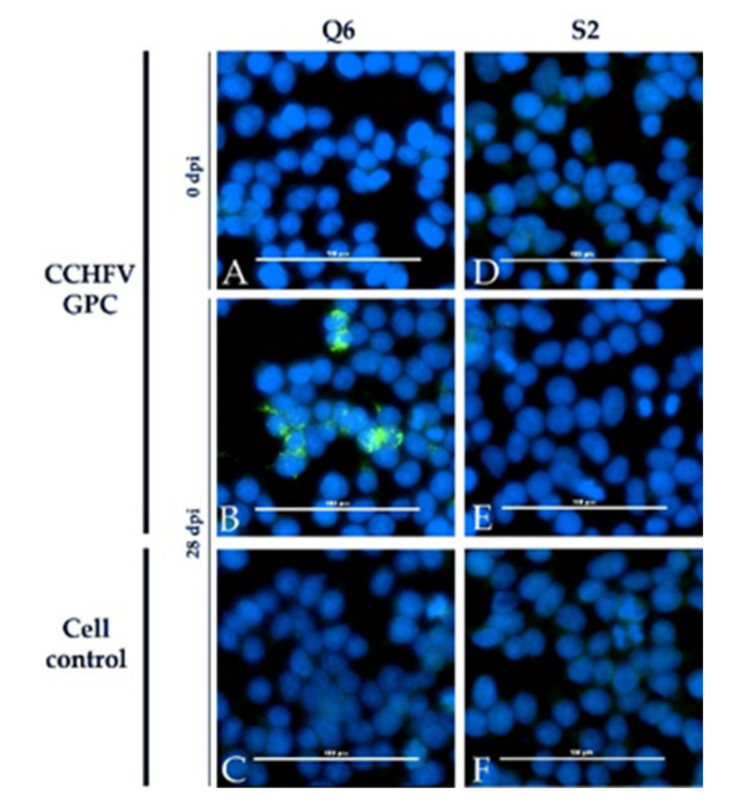
Euroimmun iIFA with cattle NSDV antisera: Results for one infected calf (Q6) are exemplarily presented together with the results for the mock control calf (S2). A specific staining is demonstrated for cells expressing CCHFV GPC (**B**). All controls (cell control: (**C**); serum prior infection: (**A**) did not lead to a positive signal. The control calf S2 did not test positive under any circumstances (**D**–**F**).

In contrast, all sera apart from the immunized sheep were clearly positive in the immunofluorescence assay (Euroimmun, Luebeck, Germany). The sheep sera led to a specific staining of cells expressing CCHFV GPC (glycoprotein precursor) and CCHFV N, whereas the bovine sera stained the CCHFV N-expressing cells only. Sera prior to infection as well as sera of the control animals did not test positive. Non-transfected control cells were also clearly negative. One example for each species is depicted (Figure 6 and Figure 7).

## 4. Discussion

Cross-reactions can lead to a decrease of the specificity of serological assays. Therefore, it is crucial to investigate the impact of cross-reactivities between related viruses, especially if these have an overlapping host and distribution range. If multiple antibodies can be detected in parallel, it is always questionable, whether these antibodies are raised due to co-infections or are rather a consequence of an immunological cross-reaction. 

**Figure 8:**
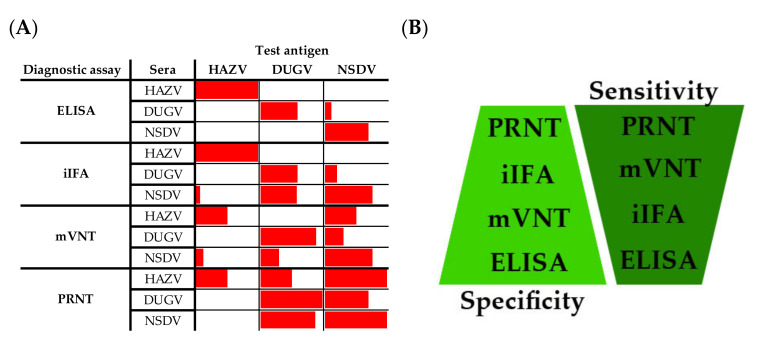
Specificity and sensitivity of HAZV, DUGV, and NSDV assays: Panel (**A**) shows the proportion of positive tested sera within a specific assay in relation to all sera following infections/immunizations with the same challenge virus, which were at least positive in one homologous virus-specific test. Exemplarily, seven sera from the DUGV studies were tested positive in the NSDV PRNT out of ten animals with DUGV antibodies detectable via any DUGV assay, leading to a bar of approx. 70% length. Panel (**B**) visualizes the ranking order of sensitivity and specificity for all diagnostic assays, with highest sensitivity achieved by PRNT and highest specificity by ELISA.

In the first part of this study, different diagnostic assays for the detection of antibodies against *Orthonairoviruses* were analyzed and cross-reactions between HAZV, DUGV, and NSDV were evaluated. Figure 8 shows an overview of the results obtained within this study. A ranking in terms of sensitivity and specificity can be established for the assays presented if not only the data obtained in this study but also the results of the single animal experiments are taken into account (Figure 8b) [18,19,20]. Exemplarily, the different ELISAs showed a reduced sensitivity compared to the other assays, as only 6 (out of 11 seroconverted) sheep were tested positive with the NSDV ELISA, and moreover the first detection of antibodies was shifted at least one sampling time point later than that measured by mVNT or iIFA. The highest sensitivity was achieved with the PRNT as for example only this assay could already demonstrate seroconversion in sheep, which were necropsied 7 days post NSDV infection [20]. The same conclusions concerning sensitivities were also drawn during the recent DUGV animal trials [19].

The indirect ELISAs based on recombinant N proteins revealed a high specificity, as only one single serum sample (DUGV-IMMU-calf-1) gave a weak positive result for a heterologous virus (NSDV). This is most likely due to the extremely high antibody titers of the immunized calf. This serum was also tested positive with the FLI CCHFV in-house ELISA [19]. Such hyperimmune sera might probably not mirror the actual antibody titers following natural infections and therefore, this result does not raise major concerns. More precisely, this serum was always diluted 1/1600 before being used in the DUGV-specific ELISA and DUGV antibody positive African cattle sera revealed at the utmost three-fold corrected OD values compared to the serum sample of DUGV-IMMU-calf-1, even though they were diluted only 1/20 [22]. This is preliminary evidence that natural infections actually do not lead to these high DUGV antibody titers. However, it remains uncertain, which maximum antibody levels can be reached following natural infections and moreover, whether these titers decrease over time. Cross-reactivities regarding hyperimmune sera were also observed in another ELISA based on recombinant DUGV N protein, when challenged with HAZV or CCHFV mouse ascitic fluids, but these findings might similarly not represent the situation in the field [23]. Consequently, we conclude that the ELISAs are generally highly specific for the detection of virus-specific antibodies.

However, this controlled study design with only defined positive sera may not reflect the actual situation when testing field samples. Non-specific binding of serum components to the ELISA plates or antigens or cross-reactive antibodies against other microorganisms can also lead to a reduced ELISA specificity.

In the indirect immunofluorescence assay (iIFA) cross-reactions were mainly observed within the same serogroup, as DUGV-infected animals were tested positive with the NSDV iIFA and vice versa. However, only one single serum dilution was tested (1/50) and a comparison of the corresponding iIFA titers against the different viruses would have probably led to the correct serological diagnosis. Nevertheless, it is current practice to evaluate just one single serum dilution in several commercial iIFA kits (e.g., Euroimmun CCHFV iIFA). A subsequent titration of the sample employing all different viruses would be quite time-consuming and moreover, the high inter-observer variability in immunofluorescence assays may influence the results.

The two neutralization assays did surprisingly also reveal cross-reactivities. In general, neutralization assays are estimated for their high specificity and the differentiation of distinct virus species should actually be easily feasible. However, DUGV and NSDV antibodies had cross-neutralizing features mainly in the PRNT. Even though the neutralization titer against the homologous viruses was higher than for the heterologous virus, an individual testing would have led to false positive results in multiple occasions. As the PRNT is very time-consuming and not suitable for high-throughput testing, this assay is a useful tool to uncover these cross-reactions under well-controlled laboratory conditions. The specificity of the mVNT is appropriate for diagnostic purposes and could even be improved, if the cut-off for positive samples would be increased (e.g., >1/15). But this would of course immediately lead to reduced sensitivity. Nevertheless, this is likely the better choice, as the majority of true positive sera will lead to significantly higher titers. The most surprising finding was that one immunized sheep (HAZV-IMMU-sheep-1) did not exhibit HAZV-neutralizing antibodies via PRNT, but in contrast lead to a reproducible reduction of plaques in the DUGV and NSDV PRNT. A presumably different receptor usage or variable attachment or internalization characteristics between these viruses may offer a possible explanation for this phenomenon. The cross-reactive antibodies may bind to epitopes—directly or via steric constraints, which are actually a target for DUGV/NSDV-neutralizing antibodies. In contrast, these epitopes may not be crucial for HAZV (and CCHFV?) infections and therefore, these cross-reactive antibodies might lack virus-specific neutralizing features for viruses of this serogroup. Another possible explanation might be the high cell-culture adaptation of the HAZV stock used in contrast to the NSDV and DUGV virus stocks. Recently, another group has performed PRNTs with CCHFV, DUGV, and HAZV and some CCHFV seropositive samples have led to a clear reduction of DUGV and HAZV plaques respectively, emphasizing again the cross-neutralizing features between these two serogroups [24].

As already indicated, the explanatory power of former cross-reactivity studies is limited and the transfer of these results is difficult (e.g., direct comparison of hyperimmune sera vs. monoclonal vs. polyclonal sera, whole virus antigen vs. recombinant proteins vs. partial recombinant proteins, in-vivo vs. in-vitro neutralization assays) [13,14,15,16,25]. We are also aware of the limitations of this present study, as not all immunized and infected animals developed similar antibody levels. For HAZV, specific post-infection sera were even not available, as sheep and bovines proved fairly resistant to experimental infections. Therefore, it is not surprising that animals with very high antibody titers (e.g., NSDV sheep P2) cross-reacted stronger in some assays than animals with very low antibody titers (e.g., all DUGV sheep).

In summary, cross-reactions do mainly occur within the Nairobi sheep disease serogroup (NSDV and DUGV). As ruminants were not susceptible to experimental HAZV inoculation, we do not assume that it plays a major role in ruminants [18]. For the serological diagnosis of NSDV and DUGV infections, it is clear that a severe disease in small ruminants might probably be due to rather a recent NSDV infection, as DUGV induced a subclinical course of infection only [19].

According to our result we recommend ELISAs as a method of choice for the discriminative approach, as these have proven to be highly specific. Moreover, ELISA testing does not require BSL 3 containment and is suitable for high-throughput screening. However, more investigations with (African) field sera are needed to reassure the claimed high specificity. Inconclusive results should always be confirmed by subsequent mVNTs.

Cross-reactivities between NSDV and CCHFV were also evaluated by running the NSDV antisera in three CCHFV ELISAs and in one immunofluorescence assay. All infection sera were tested negative with the three ELISAs—re-confirming an indeed high specificity of these assays. The hyperimmunized calf was reactive, however, which might be due to higher N protein-specific antibody levels (NSDV N protein being the main immunogen in the vaccine and CCHFV N protein antigen in the CCHFV ELISAs). Cross-reactions, albeit at low levels, have also been observed recently by another group, when they tested their In-house N protein-based CCHFV ELISA with a NSDV hyperimmune mouse serum [26].

The immunofluorescence assay (Euroimmun) seems to be quite non-specific as all infected animals and the immunized calf were clearly tested positive. Whereas the calves only reacted with cells expressing CCHFV GPC (like already shown for DUGV antisera [19]), sheep antisera also reacted with the CCHFV N protein.

## 5. Conclusions

In this project, serological cross-reactions between related viruses of the genus *Orthonairovirus* were analyzed. In the first part of the study, the serological discrimination of antibodies directed against HAZV, DUGV, and NSDV was investigated, whereas in the second part of the study cross-reactions between NSDV and CCHFV were also addressed. Appropriately, ELISAs revealed high specificities in both studies and can be used more confidentially during future field studies. In contrast, immunofluorescence assays were less specific and therefore, positive test results should always be confirmed by additional assays (e.g., ELISA, mVNT).

Finally, we recommend—in line with the suggestions of the OIE Terrestrial Manual for diagnostic tests [27]—the inclusion of antisera against related viruses into the validation process of any serological assay. Only serum panels containing also antisera against related viruses will allow to determine the true diagnostic specificity.

## Figures and Tables

**Table 1:** Ruminant sera used in this study.

Viruses			
Virus	HAZV	DUGV	NSDV
Isolate	JC280	IBAR1792	IG619
Immunizations (sc. inoculation)			
Immunized animals	HAZV-IMMU-calf-1 HAZV-IMMU-sheep-1	DUGV-IMMU-calf-1 DUGV-IMMU-sheep-1	NSDV-IMMU-calf-1 NSDV-IMMU-sheep-1
Infection Trials (sc. infection)			
Infected Animals *(internal animal codes)*	**14 sheep**	**5 sheep**	**14 sheep**
	12 *challenged sheep* *A1-6, B1-6;* *two mock controls C1, C2*	*four challenged sheep* *G5, G6, H5, H6;* *one mock control I2*	12 *challenged sheep* *N1-4, M1-4, P1-4;* *two mock controls O1, O2*
	**5 calves**	**5 calves**	**5 calves**
	*four challenged calves* *D5, D6, E5, E6;* *one mock control F2*	*four challenged calves* *J5, J6, K5, K6;* *one mock control L2*	*four challenged calves* *Q5, Q6, R5, R6;* *one mock control S2*
Course of infection	Sheep and cattle are not susceptible	Subclinical infection in sheep and bovines	Severe disease in sheep Subclinical infection in bovines
Sero-conversion	No	Yes bovines > ovines	Yes ovines > bovines
Reference	[18]	[19]	[20]

**Table 2:** Serological assays.

Diagnostic Assay	Serum Dilution	Test Criterion for Positive Results
Indirect ELISA	1/20	Corrected OD > ELISA cut-off
iIFA	1/50	Specific fluorescence signal compared tonon-infected control cells
mVNT	1/5 + log 2 dilutions	mVNT titers ≥ 1/7
PRNT	1/8 + log 2 dilutions	PRNT_80_ titers ≥ 1/8

**Table 3:** Results obtained with CCHFV assays and NSDV antisera.

Diagnostic Assay incl. Antigen		ELISA			iIFA
		**Vector-Best**	**IDVet Double Antigen**	**In-House**	**Euroimmun**
		Inactivated whole virus, clade IV	Recombinant N protein, clade III	Recombinant N protein, clade V	Transfected cells with GPC and N protein, clade III
Serum	Immunized calf	Positive	Positive	Positive	Positive
	Immunized sheep	*	Negative	*	Negative
	Infected calves	Negative	Negative	Negative	Positive
	Infected sheep	Negative	Negative	Negative	Positive

* The post immunization serum led to high non-specific reactions.

## Data Availability

The data presented in this study are available within this manuscript Hartlaub et al., Microorganisms.

## Supplementary Materials

The following are available online at https://www.mdpi.com/article/10.3390/microorganisms9071493/s1. Table S1: Results cross-reactivity mVNT: HAZV, DUGV, NSDV. Table S2: Results cross-reactivity PRNT: HAZV, DUGV, NSDV
