# Supplementary material for: Deciphering Antibody Responses to Orthonairoviruses in Ruminants
Source: Microorganisms. 2021 Jul 13;9(7):1493. doi: 10.3390/microorganisms9071493 (PMC8303759; doi:10.3390/microorganisms9071493)
Supplement: Supplementary file 1 [file microorganisms-09-01493-s001.zip › microorganisms-1285320-supplementary.pdf]

Table S1: mVNT cross-reactivity results: HAZV, DUGV, NSDV

Virus	Animal	mVNT titer		
		**HAZV**	**DUGV**	**NSDV**
**HAZV**	HAZV-IMMU-calf-1	Negative	Negative	Negative
	HAZV-IMMU-sheep-1	**7**	Negative	**15**
	Infected sheep	Negative	ND	ND
	Infected cattle	Negative	ND	ND
**DUGV**	DUGV-IMMU-calf-1	Negative	**99**	**24**
	DUGV-IMMU-sheep-1	Negative	**47**	Negative
	G5	Negative	**21**	Negative
	G6	Negative	**12**	Negative
	H5	Negative	**8**	Negative
	H6	Negative	**30**	Negative
	I2 (control)	Negative	Negative	Negative
	J5	Negative	**26**	**13**
	J6	Negative	**7**	Negative
	K5	Negative	**37**	**12**
	K6	Negative	Negative	Negative
	L2 (control)	Negative	Negative	Negative
**NSDV**	NSDV-IMMU-calf-1	Negative	Negative	**387**
	NSDV-IMMU-sheep-1	Negative	Negative	**7**
	N1	**7**	**12**	**233**
	N2	Negative	**13**	**41**
	N3	**8**	**9**	**295**
	N4	Negative	**16**	**163**
	M1	Negative	Negative	Negative
	M2	Negative	Negative	Negative
	M3	Negative	Negative	Negative
	M4	Negative	Negative	Negative
	P1	Negative	**7**	**226**
	P2	Negative	Negative	**643**
	P3	Negative	Negative	**264**
	P4	Negative	Negative	Negative
	O1 (control)	Negative	Negative	Negative
	O2 (control)	Negative	Negative	Negative
	Q5	Negative	Negative	**31**
	Q6	Negative	Negative	**15**
	R5	Negative	Negative	**44**
	R6	Negative	Negative	**11**
	S2 (control)	Negative	Negative	Negative

ND: not determined
Negative: <7

Table S2: PRNT cross-reactivity results: HAZV, DUGV, NSDV

Virus	Animal	PRNT80 titer		
		**HAZV**	**DUGV**	**NSDV**
**HAZV**	HAZV-IMMU-calf-1	**8**	Negative	**8**
	HAZV-IMMU-sheep-1	Negative	**32**	**32**
	Infected sheep	Negative	ND	ND
	Infected cattle	Negative	ND	ND
**DUGV**	DUGV-IMMU-calf-1	Negative	**384**	**8**
	DUGV-IMMU-sheep-1	Negative	**512**	**16**
	G5	Negative	**32**	Negative
	G6	Negative	**12**	Negative
	H5	Negative	**16**	Negative
	H6	Negative	**64**	**8**
	I2 (control)	Negative	Negative	Negative
	J5	Negative	**256**	**64**
	J6	Negative	**24**	**8**
	K5	Negative	**384**	**64**
	K6	Negative	**48**	**8**
	L2 (control)	Negative	Negative	Negative
**NSDV**	NSDV-IMMU-calf-1	Negative	**48**	**1024**
	NSDV-IMMU-sheep-1	Negative	**12**	**32**
	N1	Negative	**320**	**1536**
	N2	Negative	**96**	**192**
	N3	Negative	**192**	**1536**
	N4	Negative	**320**	**2048**
	M1	Negative	**8**	**32**
	M2	Negative	Negative	**16**
	M3	Negative	**8**	**32**
	M4	Negative	Negative	**16**
	P1	Negative	**96**	**1536**
	P2	Negative	**384**	**4096**
	P3	Negative	**128**	**2048**
	P4	Negative	Negative	Negative
	O1 (control)	Negative	Negative	Negative
	O2 (control)	Negative	Negative	Negative
	Q5	Negative	**16**	**192**
	Q6	Negative	**48**	**48**
	R5	Negative	**128**	**256**
	R6	Negative	**24**	**96**
	S2 (control)	Negative	Negative	Negative

ND: not determined
Negative: <8
